# Body Composition and Pectoralis Major Muscle Evaluation in Women Undergoing Breast Cancer Surgery: A Longitudinal Preliminary Observational Study

**DOI:** 10.3390/muscles5010016

**Published:** 2026-02-17

**Authors:** Giulia Bongiorno, Nicole Salvador, Samuele De Cecco, Helena Biancuzzi, Francesca Dal Mas, Chiara Pinzini, Luca Miceli

**Affiliations:** 1 Friuli Riabilitazione Rehabilitation Center, 33080 Roveredo in Piano, Italy; 2 Department of Medicine, University of Perugia, 06121 Perugia, Italy; 3 Department of Medicine, University of Udine, 33100 Udine, Italy; 4 Department of Economics, Ca’ Foscari University of Venice, 30121 Venice, Italy; 5 Department of Management, Ca’ Foscari University of Venice, 30121 Venice, Italy; 6 Centro di Riferimento Oncologico, 33081 Aviano, Italy

**Keywords:** breast cancer, sonoelastography, rehabilitation, pectoralis major muscle

## Abstract

Background: The aim of this observational preliminary study is to detect any changes in body mass, muscle strength and characteristics of the pectoralis major muscle in women who have undergone breast surgery treatments. Methods: Instrumental assessments, completed before surgery and after 60 and 120 days, included sonoelastography, dynamometric examination and surface electromyography (sEMG) of the pectoralis major muscle, hand grip test, body bioimpedance analysis; the DASH (Disability of the Arm, Shoulder and Hand) questionnaire and pain assessment using the NRS (Numerical Rating Scale). Results: An initial increase in weight and fat mass was observed, followed by a reduction related to the resumption of physical activity stimulated by physiotherapy and medical support. The IC (intracellular)/EC (extracellular) ratio showed an increase in extracellular fluids in the final phase, indicative of possible water retention and early oedema. Muscle strength and DASH scores showed a functional decline, which may be explained by reduced physical activity and the direct involvement of the pectoral muscle in surgical and radiotherapy procedures. Sonoelastography showed color variations suggestive of changes in tissue stiffness, useful for distinguishing between reinforcement processes and possible scarring. Conclusions: This multidimensional approach can be useful in the early monitoring of some tissue alterations (i.e., fat mass) as an aid to define personalized rehabilitation protocols for women who have undergone breast surgery.

## 1. Introduction

### 1.1. Breast Cancer

Breast cancer is the most frequently diagnosed neoplasm among women worldwide and is also the leading cause of oncological death in the female population [1]. The highest incidence is found in Australia, New Zealand, Northern Europe and North America and the lowest in South Asia, Central and East Africa; however, the mortality rate is highest in countries such as Melanesia, Polynesia and West Africa, where more than half of affected women die from breast cancer [2]. Breast cancer treatments carry distinct long-term complications and potential disabilities. Surgery, such as lumpectomy or mastectomy with lymph node dissection, risks lymphedema causing chronic arm swelling and reduced mobility, chronic pain at the site, and body image issues leading to psychological distress. Radiation therapy effectively targets residual cells post-surgery but heightens chances of fibrosis, heart or lung damage in left-sided cases, secondary cancers years later, and persistent fatigue impairing daily function. Chemotherapy, using agents like anthracyclines and taxanes, often induces permanent neuropathy with numbness and weakness, cardiotoxicity risking heart failure, infertility, early menopause, and cognitive decline known as “chemo brain” affecting memory and work capacity. Hormone therapy with tamoxifen or aromatase inhibitors disrupts bone health via osteoporosis, triggers severe menopausal symptoms like hot flashes and joint pain, increases endometrial cancer risk, and elevates cardiovascular events, all contributing to reduced quality of life. Targeted therapies for HER2-positive cases, such as trastuzumab, and immunotherapies for triple-negative types, can cause enduring cardiac dysfunction, thyroid disorders, autoimmune conditions, and neuropathy, sometimes require ongoing monitoring and limit physical endurance [1,2]. In Italy, there has been a slight increase in cases of breast cancer, going from 82.4 to 86.5 cases per 100,000 inhabitants (both male and female) [3]. In 2018, five-year survival from diagnosis in young Italian women reached approximately 95% [4], confirming the importance of lifestyle [5,6,7,8], early diagnosis and the expansion of screening programs, such as the anticipation of mammography to the 45–49 age group already active in some Italian regions [9,10,11]. However, the prolongation of survival leads to addressing the issue of quality of life, which can be assessed through validated questionnaires: the DASH (Disabilities of the Arm, Shoulder and Hand) questionnaire is often used to evaluate upper limb functionality in clinical practice, allowing identification of motor restrictions and the impact on quality of life in breast cancer patients [12,13]. Radiotherapy acts significantly on the growth and repair capacity of the muscle, causing direct damage to muscle fibers and satellite cells, which can be identified through sonoelastography, an advanced ultrasound technique that allows one to quantify the stiffness of soft tissues, i.e., shoulder mechanical rigidity. In this context, Shear Wave Elastography (SWE) represents a useful tool capable of early monitoring of the long-term effects of treatments at the tissue level, quantifying the volume of tissue involved and thus allowing timely referral of the patient towards a targeted rehabilitation program. In previous reports, the evidence shows how, already within the first 12 months after completion of radiotherapy, the pectoralis major muscle presents a significant increase in stiffness, indicative of early fibrosis processes, with a progressive trend especially in patients exposed to medium doses ≥ 40 Gy [14,15,16].

These effects can compromise performance in daily activities and, in the long term, increase the risk of developing subacromial impingement or adhesive capsulitis [14,17].

### 1.2. Surface Electromyography

Surface electromyography (sEMG), a non-invasive diagnostic method that records muscle electrical activity through the cutaneous surface electrodes, can be useful in the study of shoulder muscle activation and coordination in healthy breast subjects and in cancer patients. sEMG, integrated with kinematics, can be an important support in personalized oncological rehabilitation because it allows understanding the compensatory strategies adopted and orienting the therapeutic exercise towards specific muscles, such as the pectoralis major [18,19]. Bongiorno et al. have recently proposed the employment of the ratio between the mean percentage of RMS (root mean square) and maximal voluntary isometric contraction (%RMS/MVIC) as a simplified and reliable indicator of muscle fatigue [20].

### 1.3. Body Composition and Strength

Oncology therapies used to treat breast cancer, particularly chemotherapy, radiation therapy, and hormone therapy, can result in long-term side effects that significantly impact the musculoskeletal and metabolic health of patients. Among them, osteoporosis, a condition characterized by reduced bone mineral density that makes bone more fragile and susceptible to fractures, represents one of the sequelae with the most relevant clinical finding [21]. Sarcopenia, defined as the reduction of muscle mass, strength and performance [22], is also frequent in breast cancer survivors and among the main etiological factors of this damage are physical inactivity, malnutrition and the side effects of chemotherapy and hormone therapy. In this context, the evaluation of muscle strength plays a central role from both a prognostic and rehabilitation point of view. Among the different tools available, the grip strength test (Handgrip Strength, HGS) represents a simple, rapid and reliable measure of forearm muscle strength, already widely used in various clinical contexts and now the subject of growing interest in oncology, particularly in women who have survived breast cancer [23]. In parallel, weight gain after the end of care, often characterized by an increase in visceral fat mass, is related to a greater risk of recurrence and specific mortality for breast cancer. It has been observed that a high body mass index, and particularly an excess of central adiposity, favors a chronic inflammatory state which, in women after menopause, leads to an increase in the production of estrogen, the main risk factor for tumors positive for estrogen receptors [24]. In this context, bioelectrical impendence analysis measurement (BIA) is a non-invasive, rapid and low-cost method widely used in clinical practice for the evaluation of body composition. In the context of breast cancer, the assessment of body composition assumes fundamental importance since some alterations caused by hormonal therapies, such as sarcopenic obesity, have been recognized as negative prognostic factors in patients with this neoplasm. Recent studies have also highlighted the need to validate the use of BIA in this population because the calculation algorithms currently used are derived from samples of healthy subjects and may not be fully accurate in cancer patients [25]. Physiotherapy represents a key intervention to counteract limitations on patients [26,27,28,29,30], osteoporosis, sarcopenia and weight gain: physical exercise, in fact, can reduce pain, increase bone mass and muscle resistance, as well as improve joint mobility and posture. It is important for the professional to use protocols favoring low or moderate intensity activities, with personalized objectives and modalities; in addition, the integration of physical activity with adequate nutritional support and with periodic monitoring of body composition allows optimizing the results and preventing long-term complications [21]. The purpose of the study was to identify anthropometric and/or instrumental variables capable of tracking over time the rehabilitation needs of women who underwent breast surgery.

## 2. Materials and Methods

### 2.1. Participants

This preliminary observational exploratory study involved five female patients undergoing elective breast surgery. Surgeries included quadrantectomy or mastectomy with or without removal of sentinel lymph node or axillary lymphadenectomy and with possible reconstruction by expander or prosthesis. All patients had also undergone, depending on the clinical situation, hormone therapy, radiotherapy and/or chemotherapy. For inclusion in the study, patients had to be 18 years of age or older, belong to ASA classes I-III, be able to provide informed consent and ensure availability to participate in periodic follow-up visits at the IRCCS CRO in Aviano. Patients with shoulder pathologies already present before breast surgery (both ipsilateral and contralateral to the neoplasm), with myalgia or previous rheumatological and peripheral neurological pathologies, with severe scoliosis or other significant pathologies in the spine were excluded. Furthermore, patients who refused to participate in the study (absence of signed informed consent), those scheduled for bilateral breast surgery, patients undergoing excisional biopsy surgery and those treated with radiotherapy or chemotherapy in the preoperative phase were excluded. All participants signed a written informed consent, previously approved by the Internal Review Board of the IRCCS CRO of Aviano and by the Single Regional Ethics Committee (CEUR) of the Friuli Venezia Giulia region. All women were taking exemestane 25 mg once daily as their sole therapy, started approximately two months (7–9 weeks) post-surgery.

### 2.2. Instruments and Protocol

**Figure 1:**
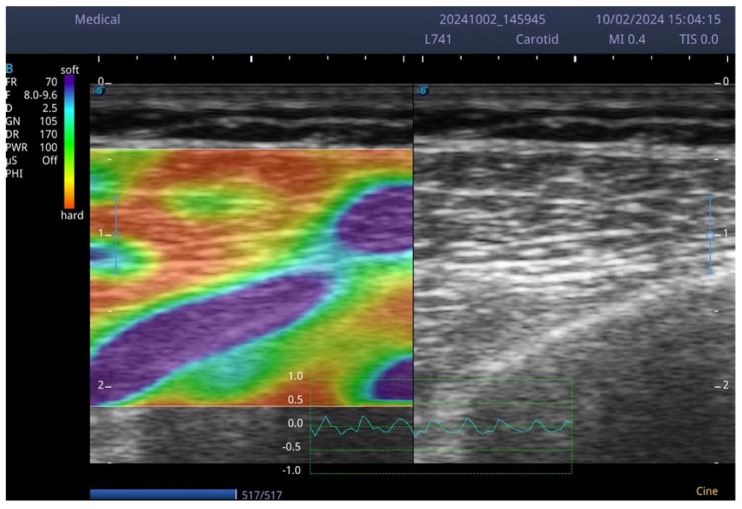
Example of sonoelastographic (SEG) image and percentages of pixels according to the color scale (Red 36%, Green 37%, Blue 26% in this image).

The various tests administered at the three predefined time points (preoperative, and 60 and 120 days post-surgery) to assess the patients (described in detail below) were divided into two categories. From an anamnestic perspective, we focused on the DASH questionnaire and on pain assessment using the NRS (Numerical Rating Scale), whereas from an instrumental perspective, we first collected data indicative of general fitness status (handgrip strength to obtain an estimate of overall force production, and bioimpedance analysis to assess whole-body and segmental body composition). Secondly, we focused on shoulder range of motion (ROM), and finally we investigated in greater detail pectoralis major muscle strength (using a dynamometer), neuromuscular fatigue of the same muscle (through surface electromyography), and, as a more cutting-edge aspect, the sonoelastographic characteristics of the pectoralis major muscle. Data described above, accompanied by the patient’s clinical history, were collected preoperatively, approximately 60 and 120 days post-surgery. Data collection form included name and surname, age, visit number, dominance, affected side, pre- or post-surgical evaluation and date of surgery, type of surgery performed, therapeutic treatments performed, results of the evaluation with the impedance scale and the following measurements performed bilaterally: Handgrip Strength Test dynamometry (Chinesport, Udine, Italy) expressed in Kg, for the strength of both pectoralis major muscles, surface electromyography to analyze fatigue of the same muscles, sonoelastography for the determination of the elasticity of the pectoralis major tissue, the Muscle Quality Index (Muscle Quality Index—hand grip strength/arm muscular mass-Baseline evaluation instruments, New York, NY, USA) expressed in Kg, NRS scale for the evaluation of shoulder, measurement of the shoulder active and passive joint ROM (range of movement) and detection of any joint limitations or lymphedema. The patient also filled out the DASH questionnaire. In the current study, the electromyographic recording was done with Bluetooth electromyograph (BTS freeEMG 1000, Bioengineering, Garbagnate Milanese, Italy) that can report muscle electrical activity in milliVolts (mV). The patient was positioned supine and the skin, in correspondence with the application area of the electrodes, carefully cleansed. Kendall Arbo 24 mm diameter disposable ECG electrodes, connected to recording probes, were then applied to the clavicular portion of the right and left pectoralis major muscle, approximately 4 cm below the clavicle, between the medial and lateral parts [31,32]. The first stage of the evaluation involved performing the MVIC, held for about 4 s on each side, during which the force developed with the dynamometer was also recorded. For the test, the upper limb was maintained with an extended elbow, flexed shoulder, and slightly intra-rotated, while the examiner stabilized the pelvis by placing the hand on the contralateral iliac crest. The task of the patient was to adduce the limb in an oblique direction towards the contralateral iliac crest, opposing the manual resistance exerted by the examiner. Subsequently, two further tests were carried out, one on each side, during which the patient maintained a contraction equal to 20% of the MVIC against dynamometric resistance (maximum voluntary isometric contraction) for 60 s, in the same way as the previous test, and the data were recorded. Finally, EMG Analyzer software (Version 1.0, BTS Bioengineering) calculated the slope of the decay line of firing frequencies: an angular value as a coefficient of the slope of the line greater than zero indicated a significant effort, a value less than zero a reduced effort. In addition, the RMS/MVIC% ratio was also calculated, an expression of the energy cost required to sustain the effort compared to that employed in the maximal contraction [20]: a high value indicated a considerable effort, a low value a reduced effort during the exercise. The Bluetooth probes had an acquisition frequency of 1000 Hz, or 1000 data per second for each of them. For the sonoelastography, the patient in the supine position underwent sonoelastographic evaluation of the left and right pectoralis major muscle (clavicular fibers). Software was integrated with a Sonoscape Exp 1 echograph (Roma, Italy), capable of analyzing static images (screenshots) and calculating the percentage value of pixels classified according to the color scale: red (R) corresponding to more rigid tissue, green (G) for intermediate values and blue (B) for softer tissue [31]. This analysis, performed by mathematical processing with Python software (Version 3.14), made it possible to obtain an objective quantification of muscle stiffness on both the right and left sides (Figure 1). Since sonoelastography lacks well-defined normative references (nomograms) and its acquisition and analysis algorithms are not yet globally standardized, we applied our method to both pectoral muscles of the patients. This allows future comparisons between the “healthy” side and the operated side. Subjects, after ≥2 h fasting, were assessed at each follow-up visit using bioelectrical impedance analysis (BIA) with the Tanita 780 MC device (Tokyo, Japan). Participants were asked to remove their outer clothing while keeping their underwear on, and to enter the approximate weight of this underwear in the “tare” field of the instrument. The patient then stepped onto the scale barefoot and with bare hands, undergoing a five-channel evaluation (respectively for right upper limb, left upper limb, right lower limb, left lower limb, and trunk). Weight, lean mass, fat mass, intra/extracellular fluids, basal metabolism, sarcopenia index, and visceral fat percentage were then recorded and reported.

### 2.3. Statistics

The Friedman test was used for statistical analysis of the data in this preliminary study; we analyzed values of each patient in three distinct times (v1, v2 and v3), with a *p*-value < 0.05 considered statistically significant.

## 3. Results

Table 1 shows the main characteristics of the population analyzed, with references to age, affected side, dominant limb, type and date of surgery.

**Figure 2:**
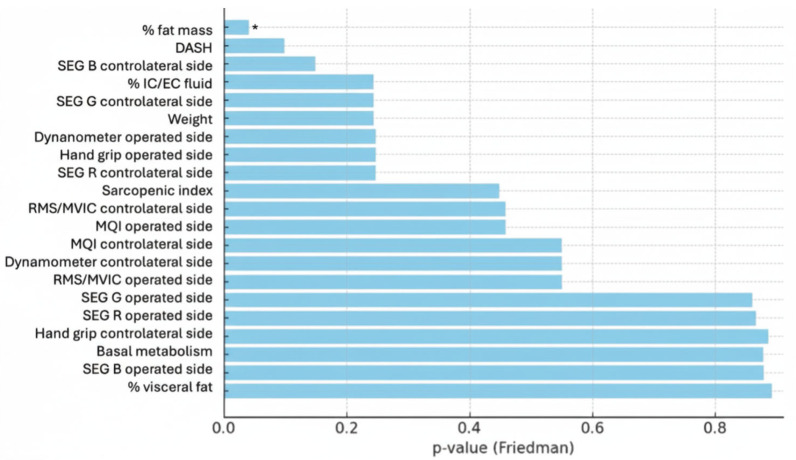
Graphical representation of the *p*-values obtained from the Friedman test for each parameter analyzed; * *p* < 0.05.

Data obtained from the evaluations conducted are presented below. Among the variables collected, only the results that have reached statistical significance at the Friedman test (*p* < 0.05) or those closer to this threshold are reported (Figure 2), as they are considered of greater relevance for the research objectives. For this study, the following were therefore taken into consideration: the percentage of fat mass, the DASH score, the three sonoelastographic values (% pixels) of the unaffected pectoralis major muscle, the percentage of intra- and extracellular fluids, body weight, the strength of the pectoralis major muscle and operated side grip strength.

Figure 2 shows the *p*-values obtained by the Friedman test for each parameter analyzed. Only % fat mass is statistically significant. Parameters located to the left, but near % fat mass, were considered arbitrarily relevant, even without reaching full significance. In contrast, the most distant values indicate variables that did not show significant differences in measurements.

**Figure 3:**
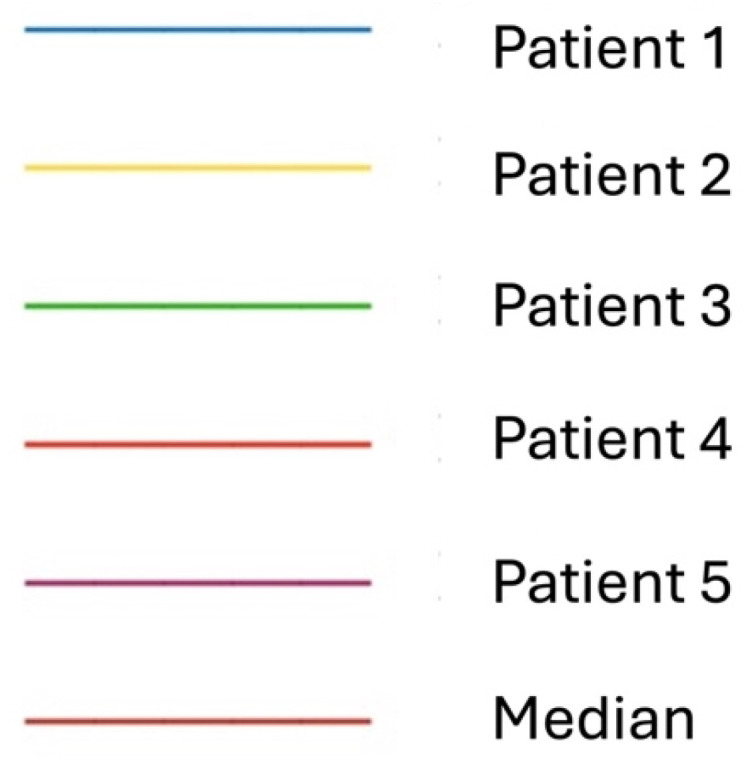
Legend for the interpretation of graphs with respect to the population analyzed.

Below (Figure 3) is an image as a reference legend for interpreting the following graphs, which summarize the data of greatest relevance for the present study.

**Figure 4:**
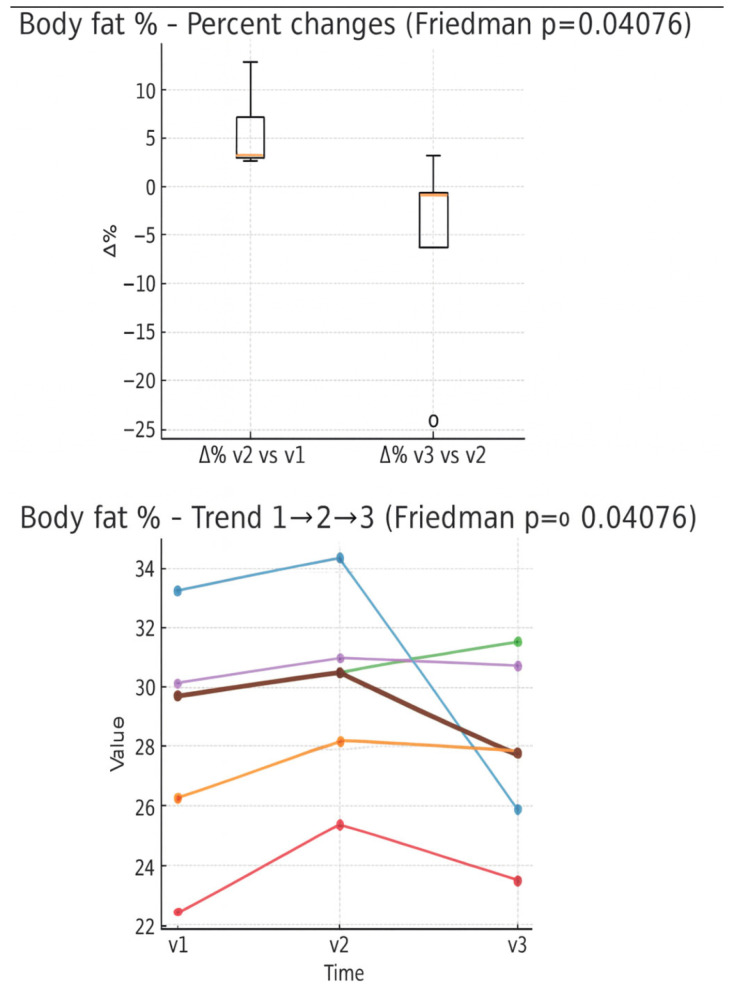
Percentage changes of % fat mass between evaluation times (boxplot) and individual trend in the three times (line plot). Expressed in percentage of body weight.

In the boxplot of percentage changes in fat mass (Figure 4), an average increase between V1 and V2 can be observed, while between V2 and V3 a percentage reduction prevails, with the presence of a marked outlier decreasing. In the individual trend graph, most patients show fat mass growth between V1 and V2, followed by a decrease between V2 and V3.

**Figure 5:**
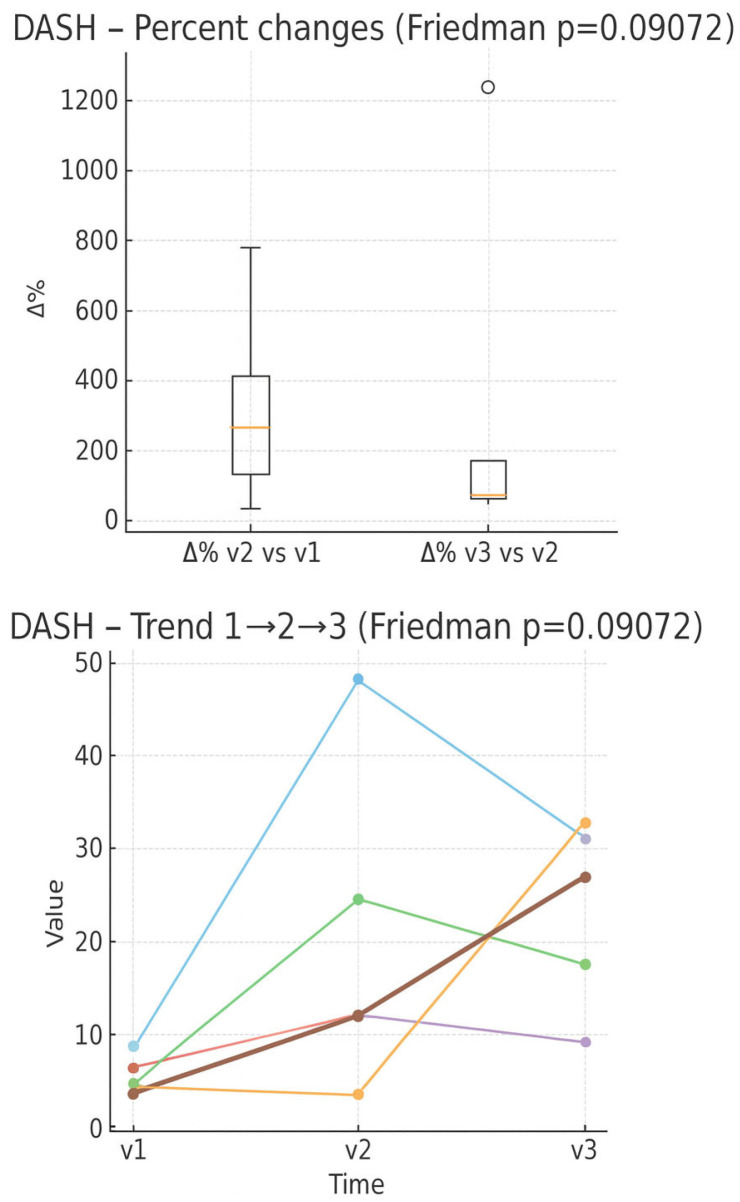
Percentage changes in DASH score between evaluation times (boxplot) and individual trend in the three times (line plot). Expressed as 0–100 percentage.

In the boxplot of percentage changes in DASH score (Figure 5), an average increase in the score is observed between V1 and V2, with wide interindividual variability, while between V2 and V3 the changes are more limited, except for one positive outlier. In the individual trend graph, however, two patients show an increase in the progressive score, the other three show a decrease.

**Figure 6:**
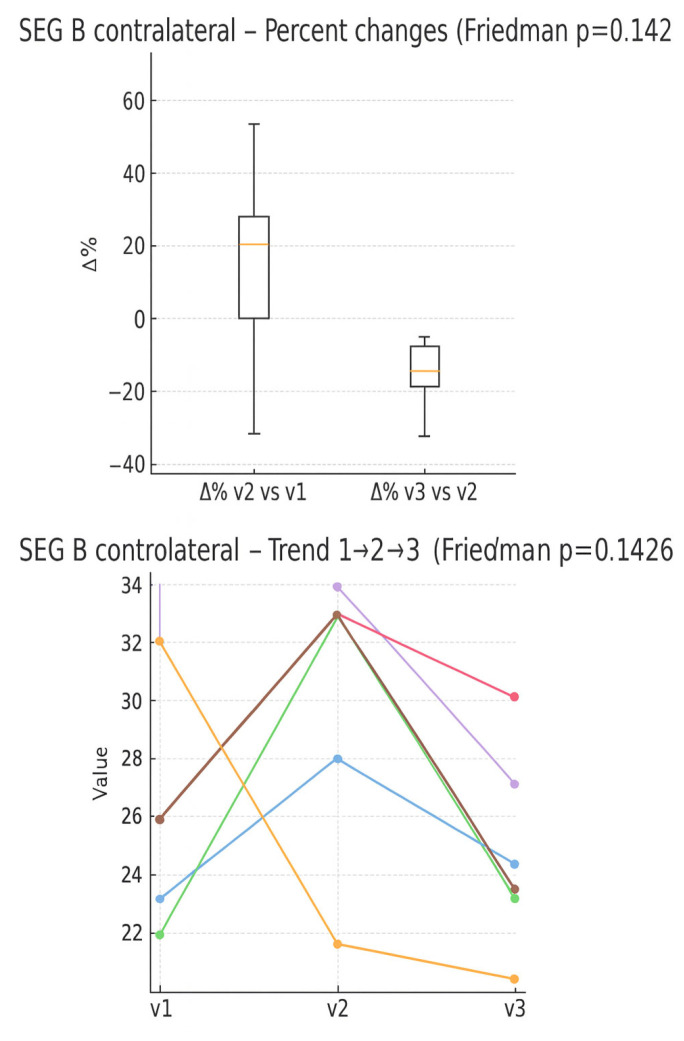
Percentage changes in the quantity of blue pixels between the evaluation times (boxplot) and individual trend in the three times (line plot). Expressed as 0–100 percentage.

In the boxplot of the percentage variations in the number of blue pixels (detected through sonoelastography) (Figure 6), an average increase is observed between V1 and V2, with wide variability and the presence of a marked outlier. In contrast, there is an average percentage reduction between V2 and V3. In the individual trend graph, several patients show an increase in value from time V1 to V2, followed by a decrease between V2 and V3.

**Figure 7:**
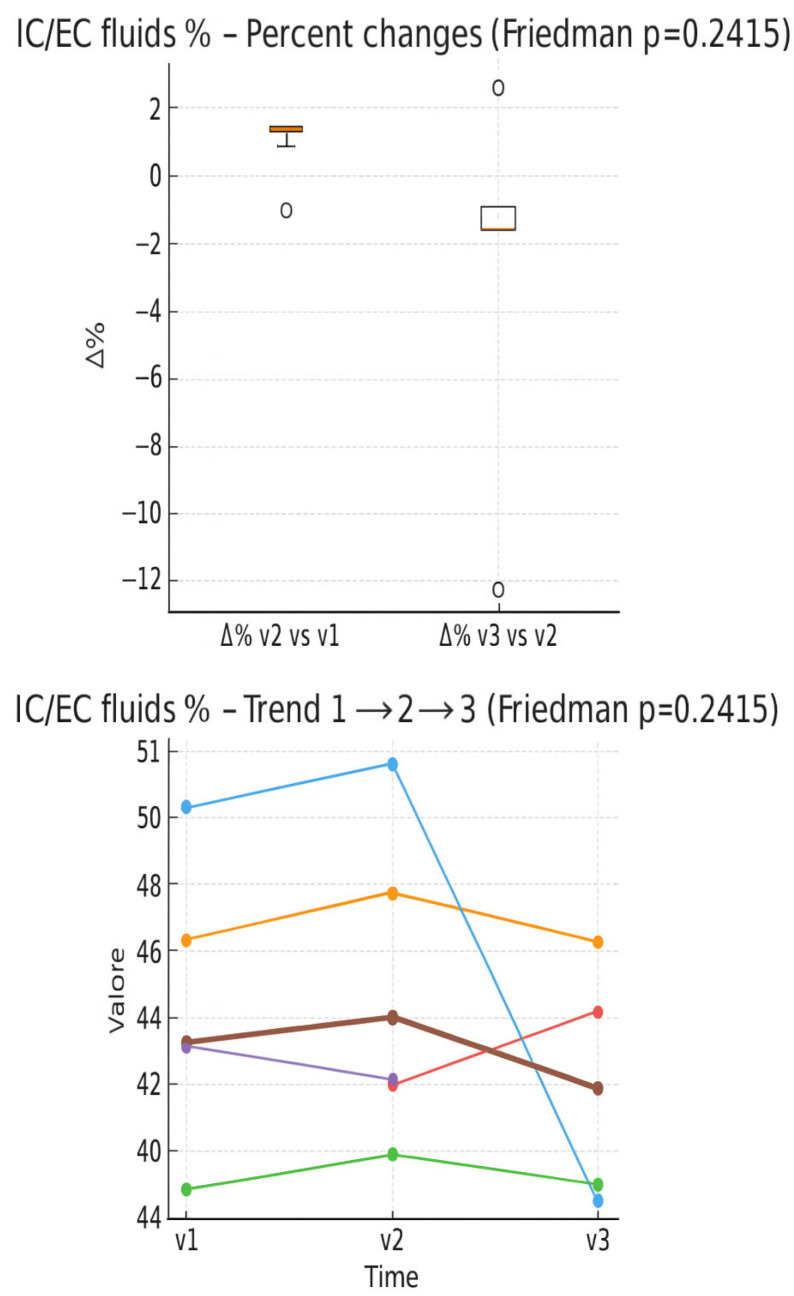
Percentage changes in the IC/EC fluid ratio between the evaluation times (boxplot) and individual trend in the three times (line plot). Expressed as 0–100 percentage.

In the boxplot of percentage changes in IC/EC fluids (Figure 7), a slight increase is observed between V1 and V2, while a slight decrease appears between V2 and V3, with outliers on both sides. In the individual trend graph, the values remain stable overall between assessment times, with some more marked subjective variations.

**Figure 8:**
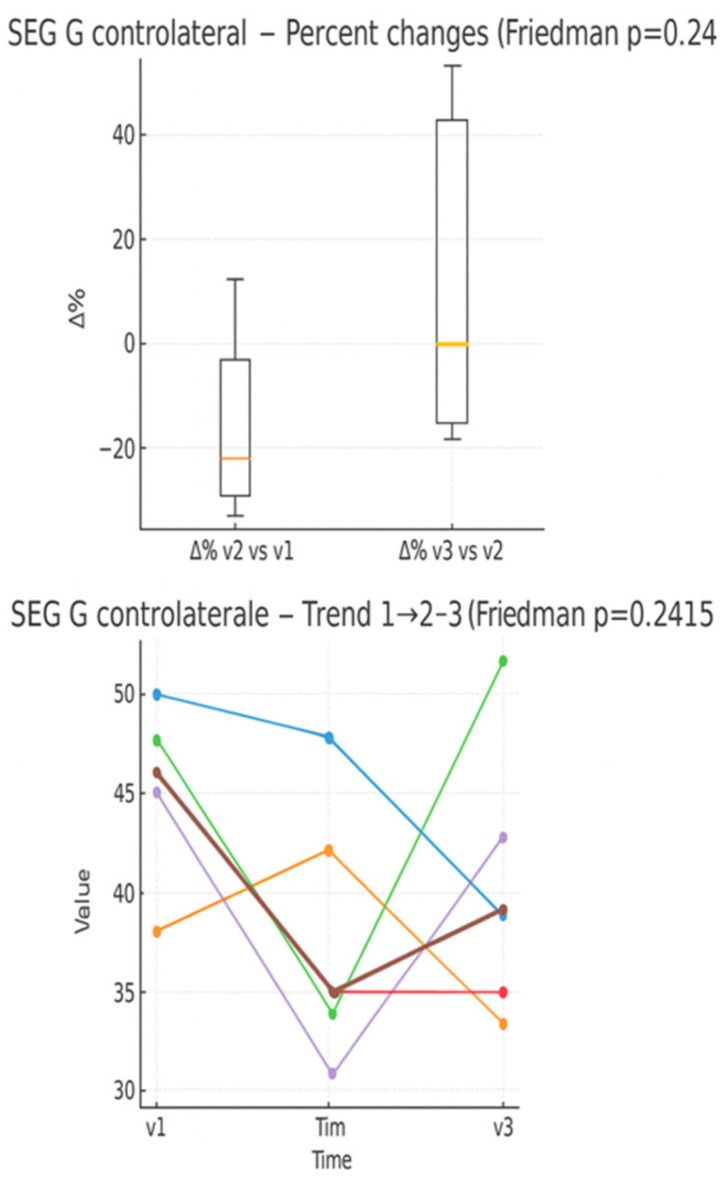
Percentage changes in the number of green pixels between the evaluation times (boxplot) and individual trend in the three times (line plot). Expressed as 0–100 percentage.

In the boxplot (Figure 8) of percentage changes in the number of green pixels, an average decrease is observed between V1 and V2, followed by an average increase between V2 and V3. In the individual trend graph, subjects show heterogeneous trajectories; some present a “U” trend, while others maintain a decreasing or relatively stable trend.

**Figure 9:**
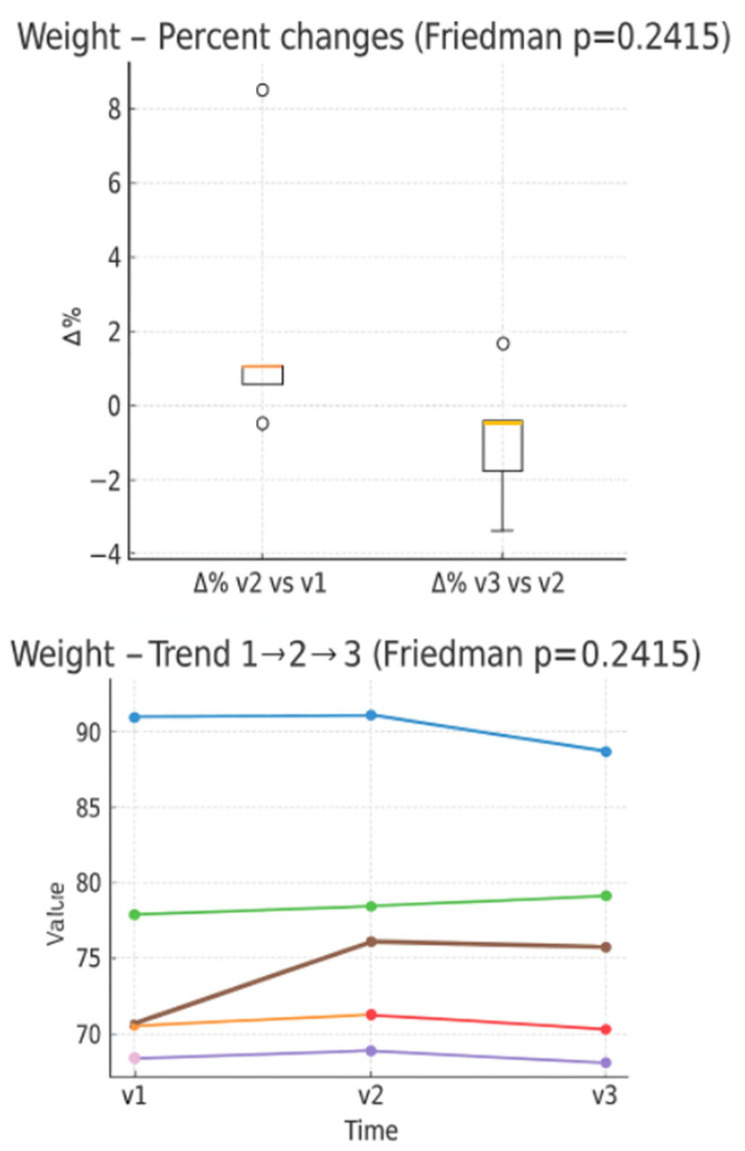
Percentage changes in weight between evaluation times (boxplots) and individual trend in the three times (line plots). Expressed in Kilos.

In the boxplot of percentage changes in weight (Figure 9), a slight average increase between V1 and V2 is observed, followed by an average reduction between V2 and V3. In the individual trend graph, values appear stable overall, with only slight fluctuations.

**Figure 10:**
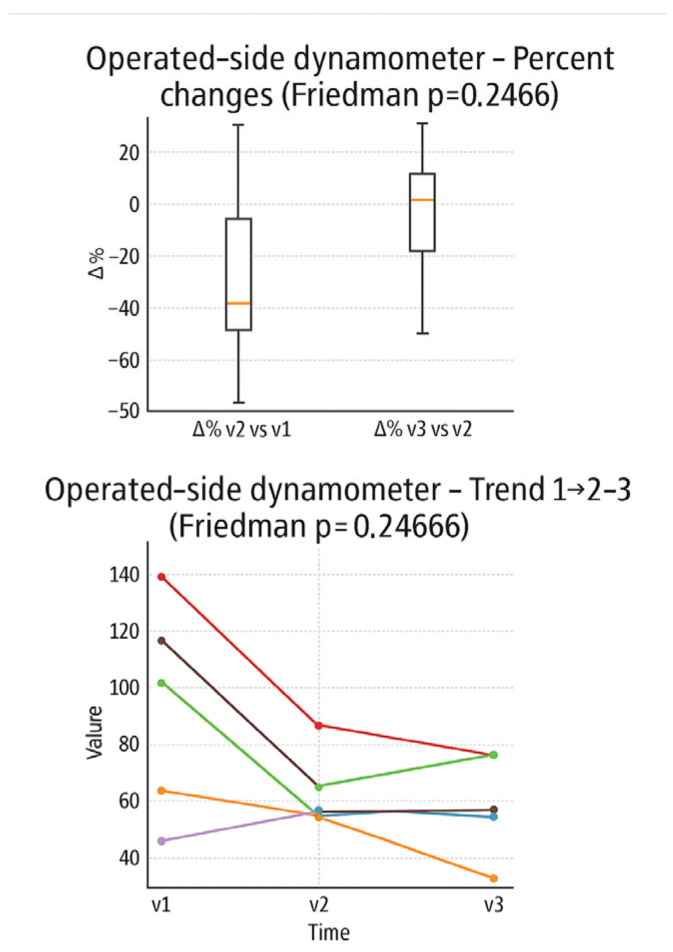
Percentage changes in dynamometer-measured force in the operated limb, between the evaluation times (boxplot) and individual trend in the three times (line plot). Expressed in Kilos.

In the boxplot of the percentage changes detected with a dynamometer (Figure 10) on the operated limb, a marked mean decrease between V1 and V2 is observed, followed by a small increase between V2 and V3. In the individual trend graph, almost all patients show a decrease in strength between V1 and V2, while between V2 and V3 some maintain stable values and others experience a partial recovery.

**Figure 11:**
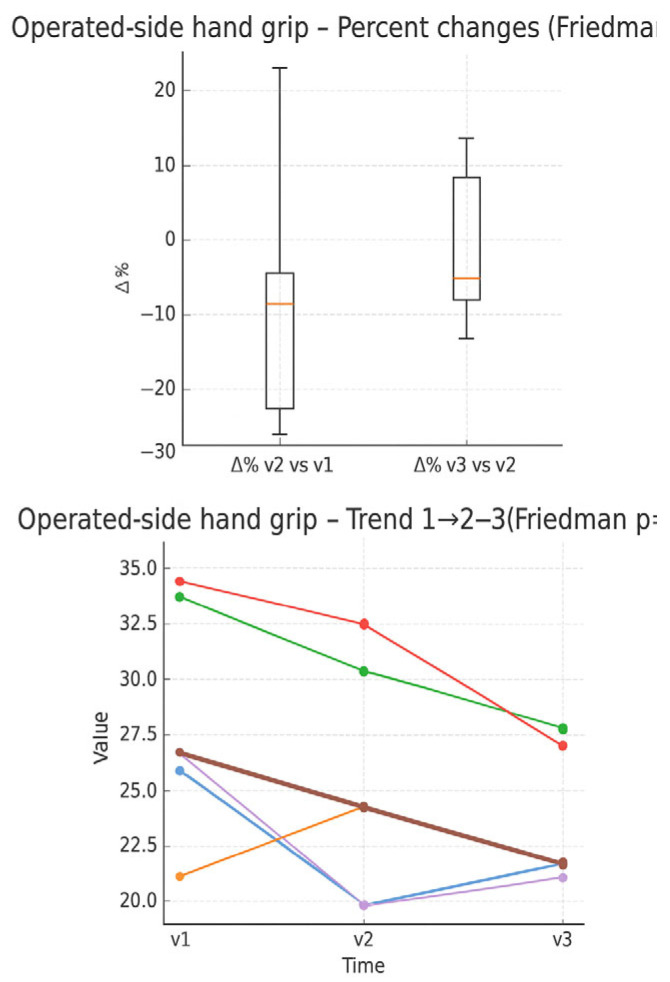
Percentage changes in HGS from the dynamometer operated side between the evaluation times (boxplot) and individual trend in the three times (line plot). Expressed in Kilos.

In the boxplot of HGS percentage changes (Figure 11) on the operated side, an average decline between V1 and V 2 is observed, followed by slight recovery between V2 and V3. In the individual trend graph, most subjects show progressive reduction in grip strength over time, with few cases showing a slight increase.

**Figure 12:**
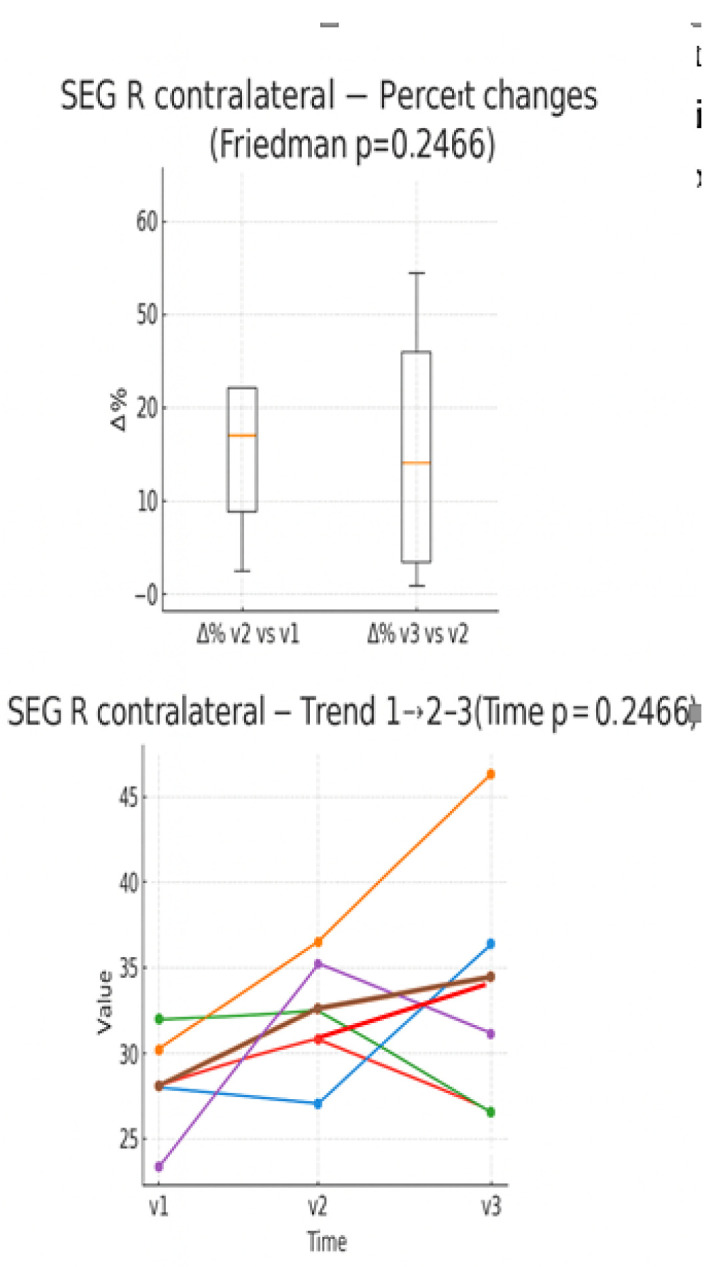
Percentage changes in the quantity of red pixels between the evaluation times (boxplot) and individual trend in the three times (line plot). Expressed 0–100 percentage.

In the boxplot of percentage variations in red pixels quantity (Figure 12), an average decrease is observed between V1 and V2, maintained between V2 and V3, although with wide interindividual variability. In the individual trend graph, most subjects show an increasing trend of the parameter from time V1 to V3.

## 4. Discussion

The study aimed to investigate a series of parameters to propose metrics to monitor over time for such patients, observing their temporal evolution and seeking consistency in their trends. Once such information was acquired, we sought to understand whether variations in certain variables had a clinical explanation related to the course of the disease and treatments, with possible positive repercussions in the future, if made available to physiotherapists, on the rehabilitation pathway of women operated on for breast cancer. The results of this Case series preliminary observation, obtained in three evaluation times (V1, V2 and V3), outline a clinical-functional picture consistent with what is reported in the literature on the course following breast surgery treatments. The only parameter that showed a statistically significant variation over time is the percentage of fat mass (*p* < 0,05), with an increase between V1 and V2 and a reduction between V2 and V3; the other parameters showed only trends, without reaching significance, but it is still useful to describe a trend characterized by an initial worsening followed by an initial functional readaptation, as expected in the first months after the intervention. A discrepancy emerged between weight stability and changes in fat mass, which increased in the early post-surgical phase (V1-V2), suggesting a loss of lean mass compensated by a relative increase in fat mass. This phenomenon can be explained by the greater sedentary lifestyle that characterizes the phase immediately following the operation, supported by pain and general weakness, despite patients being encouraged to carry out aerobic activity and specific exercises for the upper limb. This aligns with sarcopenia pathophysiology in breast cancer survivors, in which physical inactivity represents one of the etiological factors, contributing not only to a reduction in physical function, but also to worse prognosis [31]. Discussion with operators (doctor and physiotherapist) contributed to sensitizing the patients with respect to their condition, promoting the resumption of regular physical activity and greater involvement in daily living activities; this intervention was also reflected in the trend of fat mass, which in the final evaluation (V3) showed a partial rearrangement. In this context, physiotherapy represents a central intervention to counteract sarcopenia and the increase in fat mass, thanks to its educational role and the possibility of structuring targeted exercise programs; integrated with adequate nutritional support and periodic monitoring by BIA, it represents a fundamental tool of rehabilitation follow-up [21]. BIA was used because, although it does not indicate in this case the variation of intra/extracellular fluids localized to the upper limb, it can be affected by exemestane intake in general terms and therefore vary based on this therapeutic action. Compared to the ratio between intra- and extracellular fluids, the values were found to be stable overall. Between the first and second evaluations, a slight increase was observed, indicative of a good hydro-electrolyte balance and the absence of edema. In the third evaluation, however, the ratio undergoes a slight reduction, suggesting a relative increase in extracellular fluids and a possible early signal of water retention and initial edema. Such variation can be attributed to the effects of adjuvant treatments (chemotherapy and radiotherapy), undertaken by all patients after the second evaluation, except for one that initiated them after surgery, but before the second evaluation. These results highlight the importance of constant monitoring via BIA to promptly detect possible predictive indicators of edema or lymphedema and early intervention that includes regular exercise programs and, if necessary, lymphatic drainage sessions. Regarding the function of the operated upper limb, the evaluations conducted with the dynamometer on the pectoralis major muscle and through the Handgrip Strength Test showed heterogeneous results, but were, overall, consistent with the clinical-functional picture after breast surgery. In both evaluations, a marked reduction in strength is observed in the immediate post-operative phase, followed, between the second and third evaluations, by a more limited decline. The Handgrip Strength Test, recognized as a general indicator of muscle strength, confirmed a common tendency of patients to reduced physical activity in the early stage, resulting in sarcopenia and significant loss of strength of the pectoralis major muscle. The scores of the DASH questionnaire are consistent with these results: between the first and second evaluations, a marked increase was recorded, indicative of greater disability in the upper limb, followed by a partial improvement in the third evaluation. Also, in this case, the data obtained were heterogeneous: some patients showed a clear worsening between V1 and V2, followed by a partial improvement at V3, while others presented a progressive increase in the score over time. Overall, these results confirm the importance of an early start of the post-operative physiotherapy process, preceded by a targeted evaluation capable of identifying muscle deficits and functional limitations of the upper limb, since it allows reducing initial muscle pain and weakness, laying the foundations for progressive reinforcement and a more rapid return to daily activities [14,23]. A key innovation introduced in this study, compared to what has been reported in the literature, concerns the use of the Python software integrated with the Sonoscape Exp 1 ultrasound, which allowed us, by means of mathematical processing, to quantify the percentage of pixels on the chromatic scale in the clavicular fibers of the right and left pectoralis major muscle. For the purposes of the study, sonoelastographic images of healthy muscle were analyzed, observing an increase in blue color signals (SEG—sonoelastography-Blue) between the first and second evaluation, followed by a subsequent decrease, while the red signal (SEG Red) showed a constantly increasing trend in the two-time intervals. To interpret these variations, it was necessary to attribute a functional meaning to the colors detected by SWE. Blue represents a soft and elastic tissue: its percentage increase can be read as an indication of worsening, as it suggests loss of muscle tone and the need for targeted physiotherapy intervention, while its reduction can be interpreted as a sign of improvement in terms of firmness and strength of the tissue. Red indicates a rigid and potentially not very elastic tissue: a percentage increase can have a double interpretation, positive if related to a process of muscle strengthening and toning, negative if associated with scarring or fibrotic phenomena. Green, indicative of intermediate elasticity, does not provide significant information if considered in isolation and should probably be interpreted in relation to the variations in the blue and red components. In view of these premises, the following possible interpretative key can be outlined: an increase in the red signal, accompanied by a reduction in blue, suggests a process of muscle strengthening and toning; conversely, the concomitant increase in red and blue indicates with greater probability the presence of fibrosis or trigger points in the muscle examined. Therefore, in the new software, which returns a precise quantitative value of the three-color components, it is sufficient to focus attention on the two extremes, represented by red and blue. In line with expectations, SWE indices on healthy pectoralis major muscle showed no significant differences over time other than a slight improvement between the second and third measurements after urging patients to perform more physical activity (increase in red with decrease in blue): this is plausible, since the literature reports that fibrotic and radiotherapy effects are located primarily on the treated side, in which an increase in stiffness and a reduction in thickness are observed within 12 months when radiation doses are medium-high [14,15,16]; so an increase in physical activity (the need for which was assessed by the healthy person who did not present bias due to surgery) probably gave small benefits. This preliminary study aimed to explore the coexistence of the health aspect and the management of physical activity in women who have undergone breast surgery, focusing on the pectoralis major muscle and its characteristics (strength, elasticity, resistance to fatigue) as well as on body composition, with the aim of integrating the known data that are used for rehabilitation purposes (shoulder range of movement, possible presence of oedema, pain) in clinical practice. A recent meta-analysis highlighted that breast cancer patients with sarcopenia have a 33% increased risk of mortality and a risk of progression/disease recurrence increased by 29% compared to non-sarcopenic patients [33] and this brings us to the topic of physical activity. A recent [34] systematic review examined 14 studies exploring pre- and post-surgery physical activity interventions for people undergoing breast cancer surgery. The analysis, including 418 participants, identified four key themes: factors promoting and preventing engagement, physical and psychological benefits, and patient recommendations. Exercise improved recovery, strength, and well-being, helped patients regain control and reduce anxiety. Barriers included fatigue, pain, misinformation, and treatment side effects. Group activities and clinician support enhanced adherence. Despite strong acceptance, preoperative programs were rare. The review recommends integrating co-designed, evidence-based physical activity interventions into standard surgical care for breast cancer patients. A Cochrane review [35] analyzed 24 randomized controlled trials (RCT) involving 2132 women treated for breast cancer. The review assessed the effectiveness and safety of exercise in preventing or improving upper-limb dysfunction, including reduced range of motion, pain, and lymphedema. Early post-operative exercise improved short-term shoulder flexion and abduction but slightly increased wound drainage volume and duration. Structured physical therapy significantly enhanced shoulder mobility and function without raising lymphedema risk. Resistance and stretching programs during or after adjuvant therapy improved strength and quality of life (QoL). Overall, exercise is safe and beneficial for restoring upper-limb mobility after breast cancer treatment. A recent systematic review and meta-analysis [36] examined 20 randomized trials (2442 women) comparing early, unrestricted shoulder range-of-motion (ROM) exercise—started within three days after breast-cancer surgery—with delayed or usual-care programs. Early exercise modestly improved shoulder flexion, particularly in recent studies, without clear increases in wound complications once modern surgical techniques were considered. Short-term lymphedema risk appeared higher, but evidence quality was low to very low. Total drainage time and hematoma incidence were slightly greater with early exercise, though not clinically relevant. Overall, early postoperative exercise seems safe and may enhance recovery, but individualized, patient-centered decisions are recommended. The association between body composition, the pectoralis major muscle, and breast cancer surgery is articulated, considering the impact of surgical and therapeutic therapies on muscle quality and on patient outcomes. Evidence highlighted the importance of body composition, particularly muscle mass and quality, in recovery and survival rates in breast cancer patients. The pectoralis major could present muscle fibrosis due to the radiation therapy, and poorer muscle quality is correlated with higher body fat percentages, with functional difficulties following surgery [37,38,39]. Sarcopenia is associated with increased mortality risk and measurements of the pectoral muscle area can be useful markers of sarcopenia [38,39]. This consideration underlines the importance of a precise and personalized medicine, especially in frail patients. Reduced muscle mass (sarcopenia) is linked to a higher risk of death in individuals with breast cancer. Reports used in this preliminary study could therefore be part of a file available to both physiotherapists and kinesiologists, to create a seamless path between healthcare and physical activity, offering patients and trainers an assessment of body composition, strength, and degree of fatigability of the pectoralis major muscle, the main player in breast surgery. Considerations expressed above have led us to also highlight the role of modern breast rehabilitation, which should keep in mind the management of physical activity in women operated on for breast cancer and not only arm recovery. In Italy, physiotherapists generally have greater knowledge and competence in the functional recovery of patients, while kinesiologists often know the topic of physical activity better, mainly in healthy subjects since theirs is not a healthcare profession [40]. However, for some years now there have been some master’s programs for kinesiologists on adapted physical activity for certain stabilized chronic conditions [41], and therefore, the time might be ready for an integration of skills between these two professions, to provide continuity of care to breast cancer patients who, after and during a hospital physiotherapy pathway, can benefit from an adequate extra-hospital physical activity pathway. The presented results are preliminary; therefore, the conclusions require further studies.

### Strengths and Limitations

The strength of this study is the adoption of a multidimensional evaluation approach, capable of integrating the main areas in which the negative post-operative consequences related to the specific oncological pathway are observed, monitored at three distinct moments within real oncological caretaking. Limitations are the small number of subjects included, which will make it necessary to perform an adequate sample size calculation for future studies to best interpret even the non-statistically significant outcomes from our initial analysis; clinical heterogeneity linked to the different adjuvant therapies undertaken, which reduced statistical power and increased variability; and lack of established standard references for the interpretation of sonoelastographic data. All these observations make this study a preliminary experience pending robust data.

## 5. Conclusions

In conclusion, although the small sample size does not allow for definitive statements but requires further investigation, given that it is a study based on a preliminary case series preparatory to further experiences [42], the results of this preliminary and innovative study highlights the importance of longitudinal monitoring, which accompanies the patient from care to the later stages, to promptly identify alterations that may compromise functionality, such as pain and stiffness, with repercussions on quality of life. It therefore appears essential to combine the monitoring of body weight with the assessment of body composition and pectoralis major muscle health status to initiate early mobilizations and reinforcement programs aimed at the shoulder girdle and to promote the adherence of patients to internationally recommended levels of exercise when needed. This preliminary study suggests that a multidimensional assessment approach may support early identification of functional and muscular changes following breast cancer surgery.

## Figures and Tables

**Table 1:** Presentation of main characteristics of the analyzed population.

	Patient 1	Patient 2	Patient 3	Patient 4	Patient 5
Age (years)	69	67	58	62	62
Affected side	Right	Left	Right	Right	Left
Dominant side	Right	Right	Right	Right	Right
Date of surgery	25 March 2025	5 March 2025	15 April 2025	11 April 2025	18 February 2025
Type of surgery	QT e ALND	QT	QT e BLS	QT e BLS	QT e BLS

(QT: quadrantectomy, BLS: sentinel lymph node biopsy, ALND: axillary lymphadenectomy).

## Data Availability

The original contributions presented in this study are included in the article. Further inquiries can be directed to the corresponding author.
